# Paw-shake response and locomotion: can one CPG generate two different rhythmic behaviors?

**DOI:** 10.1186/1471-2202-13-S1-P70

**Published:** 2012-07-16

**Authors:** Alexander N Klishko, David Cofer, Gennady Cymbalyuk, Donald H Edwards, Boris I Prilutsky

**Affiliations:** 1 Center for Human Movement Studies, School of Applied Physiology, Georgia Institute of Technology, Atlanta, GA, USA; 2 Neuroscience Institute, Georgia State University, Atlanta, GA, USA

## 

Rhythmic limb movements like locomotion or paw-shake response are controlled by network of spinal circuits, known as central pattern generators (CPGs), as evidenced from locomotor-like and paw-shake like activity in limb peripheral nerves elicited in decerebrate or spinal animals with blocked neuromuscular transmission [4]. Unlike fictive locomotion and scratch, that are likely controlled by distinct CPGs [3], fictive paw-shake response has not been systematically investigated and it is not known whether it is controlled by a specialized CPG or by the CPG that also controls locomotion. In-vivo recordings of paw-shake motor patterns elicited by stimulation of paw skin afferents [7] have revealed high frequency hindlimb oscillations (~10 Hz) with atypical muscle synergies – reciprocal activation of anterior and posterior hindlimb muscles in each half of the paw-shake cycle; both anterior and posterior muscle groups include flexor and extensor muscles. We asked whether a paw-shake response with the atypical muscle synergies can be generated by a typical half-center locomotor CPG reciprocally activating flexor and extensor muscles.

Using software AnimatLab [2] we developed a 5-segment cat hindlimb model with 12 Hill-type muscle actuators controlled by (1) a half-center CPG activating flexor and extensor muscles (two-joint muscles received both flexion- and extension-related signals [5,6]) and (2) proprioceptive input originated from the muscle spindle and Golgi tendon organ afferents. The CPG was modeled by two single-compartment spiking neurons in a half-center configuration. Other neurons (Ia-afferents, alpha-motor neurons, Ia-interneurons, and interneurons mediating autogenic and heterogenic reflex pathways) were modeled as non-spiking neurons (firing rate model based on work by [1]). Model parameters were adjusted such that computer simulations reproduced the recorded paw-shake mechanics and the anterior-posterior muscle activation patterns.

The obtained results demonstrated that a half-center locomotor CPG can produce movement mechanics and muscle activity patterns typical for paw-shake responses if (1) the locomotor CPG is capable to operate at frequencies 3 to10 times higher than during locomotion and (2) synaptic weights in spinal circuits can be modified during paw-shake response. We speculate that the two conditions can be realized by sensory input from paw skin afferents.
